# Lymphocyte Kv1.3-channels in the pathogenesis of chronic obstructive pulmonary disease: novel therapeutic implications of targeting the channels by commonly used drugs

**DOI:** 10.1186/s13223-016-0168-3

**Published:** 2016-11-29

**Authors:** Itsuro Kazama, Tsutomu Tamada

**Affiliations:** 1 Department of Physiology, Tohoku University Graduate School of Medicine, Seiryo-cho, Aoba-ku, Sendai, Miyagi Japan; 2 Department of Respiratory Medicine, Tohoku University Graduate School of Medicine, Sendai, Japan

**Keywords:** Chronic obstructive pulmonary disease (COPD), Over-activated T-lymphocytes, Delayed rectifier K^+^-channels (Kv1.3), Therapeutic implications for COPD, Kv1.3-channel inhibitor

## Abstract

In patients with chronic obstructive pulmonary disease (COPD), over-activated T-lymphocytes produce pro-inflammatory cytokines and proliferate in situ in the lower airways and pulmonary parenchyma, contributing substantially to the pathogenesis of the disease. Despite our understanding of the molecular mechanisms by which lymphocytes are activated, we know little about the physiological mechanisms. T-lymphocytes predominantly express delayed rectifier K^+^-channels (Kv1.3) in their plasma membranes and these channels play crucial roles in inducing the lymphocyte activation and proliferation. In the pathogenesis of chronic inflammatory diseases, such as chronic kidney disease (CKD) or inflammatory bowel disease (IBD), these channels, which are overexpressed in proliferating lymphocytes within the inflamed organs, are responsible for the progression of the diseases. Since the over-activation of cellular immunity is also mainly involved in the pathogenesis of COPD, this disease could share similar pathophysiological features as those of CKD or IBD. From a literature review including ours, it is highly likely that the Kv1.3-channels are overexpressed or over-activated in T-lymphocytes isolated from patients with COPD, and that the overexpression of the channels would contribute to the development or progression of COPD. The involvement of the channels leads to novel therapeutic implications of potentially useful Kv1.3-channel inhibitors, such as calcium channel blockers, macrolide antibiotics, HMG-CoA reductase inhibitors and nonsteroidal anti-inflammatory drugs, in the treatment of COPD.

## T-lymphocytes in the pathogenesis of COPD

Chronic obstructive pulmonary disease (COPD) is one of the leading causes of morbidity and mortality in the world, due to increased cigarette smoking, environmental exposure to air pollution particles and occupational exposure to various types of dust and fumes [1, 2]. This disease is histopathologically characterized by chronic inflammation of the airways, with large numbers of inflammatory leukocytes, such as lymphocytes, neutrophils, eosinophils, macrophages and mast cells, infiltrating into the central or peripheral airways [3–7]. These inflammatory cells initially trigger the inflammatory response in the bronchi or bronchioles, which subsequently leads to fibrosis in the smaller airways and alveolar destruction, thus contributing to the pathogenesis of the disease [4]. Of note, in patients with COPD, a prominent increase was found in the total numbers of T-lymphocytes and their production of pro-inflammatory cytokines, such as interferon-γ and tumor necrosis factor (TNF), in the airways and lung parenchyma [8]. Among T-lymphocytes, CD8^+^ cells, which are more predominant than CD4^+^ cells in COPD patients, have the capacity to cause cytolysis and apoptosis of alveolar epithelial cells through the release of perforins, granzyme-B and TNF [9]. Therefore, the increased number of T-lymphocytes and their activity actually correlated well with the severity of airway obstruction and the amount of alveolar destruction in COPD [4].

## Involvement of lymphocyte Kv1.3-channels in chronic inflammatory diseases

T-lymphocytes, such as CD4^+^ and CD8^+^ cells, predominantly express delayed rectifier K^+^-channels (Kv1.3) in their plasma membranes [10, 11]. Patch-clamp studies revealed that the channels play crucial roles in facilitating the calcium influx necessary to trigger the lymphocyte activation and proliferation [11, 12]. In pathological conditions, such as in chronic kidney disease (CKD) or inflammatory bowel disease (IBD), the channels were overexpressed in proliferating lymphocytes within the inflamed kidneys or intestines, and the over-activation of the channels largely contributed to the progression of the diseases [13–15]. Previously, we demonstrated in an animal study that the overexpression of Kv1.3-channels in lymphocytes was deeply associated with their in situ proliferation in kidneys and the progression of chronic renal failure (CRF) [13]. In this study, we further demonstrated that margatoxin, a selective inhibitor of the Kv1.3-channels [16], actually ameliorated the progression of renal fibrosis in rat models with advanced CRF [13]. Along with COPD, CKD and IBD are also nowadays categorized as chronic inflammatory diseases, in which chronic inflammation or the overstimulation of cellular immunity is responsible for the pathogenesis [17]. Therefore, these diseases could share similar pathophysiological features as those of COPD.

## Involvement of lymphocyte Kv1.3-channels in COPD

**Fig. 1:**
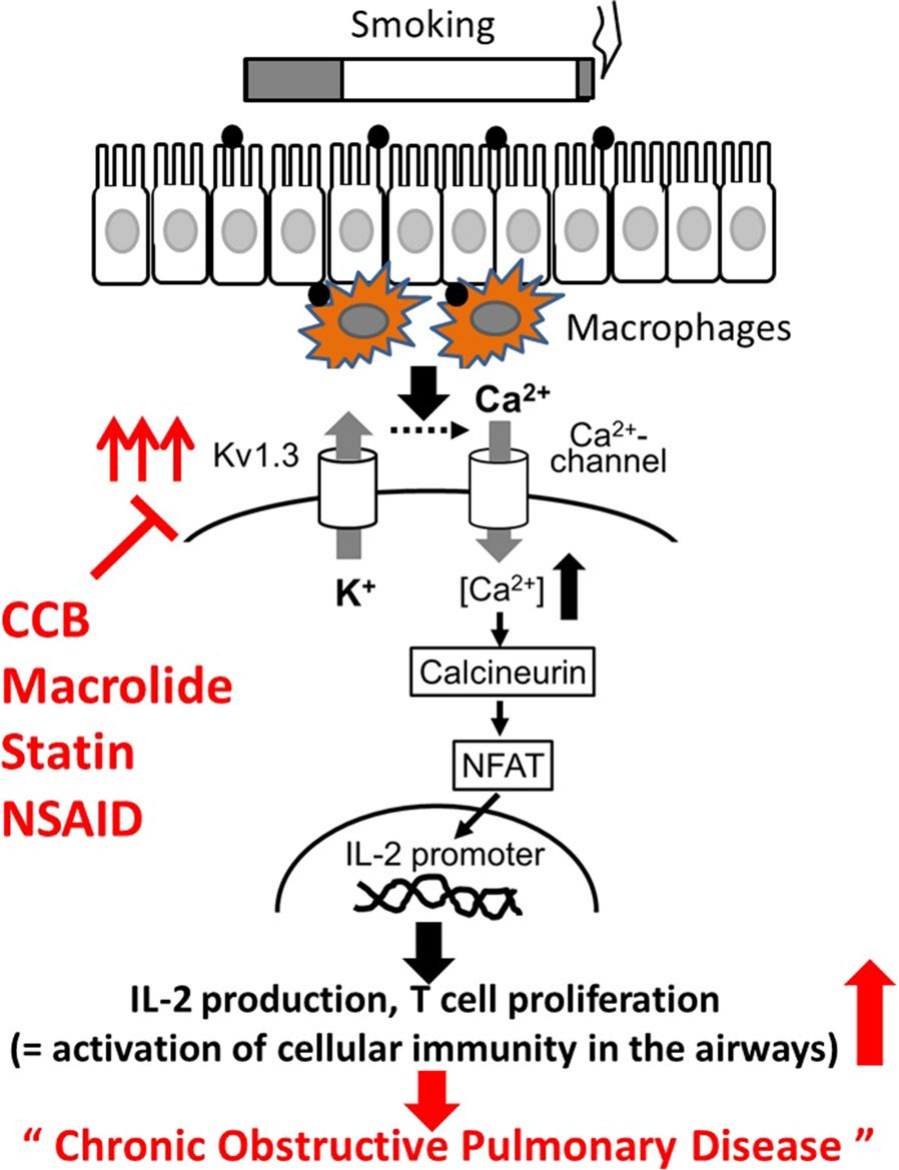
Roles of Kv1.3-channels in the activation pathway of cellular immunity in the respiratory tract and the development of chronic obstructive pulmonary disease (COPD). In the initiation of mucosal immunity in the airways, bronchial or alveolar macrophages, which phagocytose smoke particles, humorally activate T-lymphocytes within the lamina propria of the respiratory mucosa. Kv1.3-channels expressed on T-lymphocytes stimulate their IL-2 production and cellular proliferation through the activation of calcineurin/NFAT signaling pathway. The overexpression of the channels over-stimulates such lymphocyte activity, which triggers the development of chronic obstructive pulmonary disease. Potential Kv1.3-channel inhibitors, such as CCBs, macrolides, statins and NSAIDs may be useful in the treatment of COPD. *NFAT* nuclear factor of activated T cells, *CCB* calcium channel blocker, *NSAID* nonsteroidal anti-inflammatory drug

Airway smooth muscle cells express various types of K^+^-channels, such as ATP-sensitive K^+^-channels and large- or intermediate- conductance Ca^2+^-activated K^+^-channels, which play roles in airway hypersensitivity and the bronchodilation [18]. Therefore, targeting these channels, previous studies revealed the therapeutic usefulness of the channel inhibitors or the openers in the treatment of COPD [19]. Recently, isolating T-lymphocytes from the airways or peripheral blood of patients with asthma, Koshy et al. demonstrated the increased expression or activity of Kv1.3-channels in these cells [20]. Using a rat model of asthma, they further demonstrated that the administration of ShK-186, a selective inhibitor of the channel, suppressed the proliferation of lymphocytes and their cytokine production, and thus actually reduced the airway hyper-responsiveness. The bronchial inflammation of asthma is characterized by the increase in CD4^+^ T-lymphocytes and their production of pro-inflammatory cytokines, while that of COPD is characterized by the predominance of CD8^+^ T-lymphocytes or macrophages [8]. However, the absolute number of CD4^+^ T-lymphocytes is also increased in the airways of COPD patients, and they are known to play roles in perpetuating the inflammatory process [21]. Additionally, recent patch-clamp studies demonstrated that the Kv1.3-channels are predominantly expressed in both types of T-lymphocytes [10]. Since the over-activation of cellular immunity and its perpetuation in the airways is responsible for the pathogenesis of COPD [8], the Kv1.3-channels in either CD4^+^ or CD8^+^ T-lymphocytes are very likely to be over-activated or overexpressed in these patients (Fig. 1).

## Potential future studies

To substantiate the hypothesis that the activity of Kv1.3 expressed in lymphocytes is important in the pathogenesis in COPD, T-lymphocytes could be isolated from the airways of COPD patients. As previously described by Pizzichini et al. [22], spontaneous or induced sputum and peripheral blood samples could be obtained from patients with COPD and from healthy volunteers. Using the isolated lymphocytes, the patch-clamp recording technique could be applied to identify the Kv1.3-channels by detecting the voltage-dependent activation and inactivation patterns characteristic to Kv1.3 [16, 23–27]. Then, using these cells, how the selective blocking of the channels by the drugs, such as margatoxin or ShK-186 [20, 28], affected the activation or proliferation of the lymphocytes could be examined in vitro. To determine the effects of these drugs on the lymphocyte activation kinetics, detailed functional analyses will be required. Possible approaches could include measurement of the cytokine production [29], leukocyte migration assay [28] and the measurement of [^3^H] thymidine incorporation into the lymphocyte DNA [20]. The proliferation of lymphocytes could be detected by either Ki-67 antibody staining or 5′-bromo-2′-deoxyuridine (BrdU) incorporation assay.

To clarify the roles of lymphocyte Kv1.3-channels in the pathogenesis of COPD, the following in vivo experiments could be conducted. Based on previous studies, mouse or rat models of COPD, such as pulmonary emphysema, can be induced by exposure to cigarette-smoke or the intra-tracheal instillation of chemical substances, such as lipopolysaccharides (LPS), cadmium chloride, nitrogen dioxide, inorganic dusts and ozone [30, 31]. Using these animal models, the expressional abundance of Kv1.3-channels in lymphocytes within the airways or lung parenchyma could histologically be examined. To reveal the actual involvement of the channels in the pathogenesis of COPD, selective inhibitors of the channels, such as margatoxin, ShK, Psora-4 or PAP-1 [13, 32–35], could therapeutically administered to the animals and quantify the production of pro-inflammatory cytokines in the airways or alveolar parenchyma. The degrees of bronchial inflammation, small airway fibrosis and alveolar destruction could also be analyzed histopathologically [36].

Cigarette smoking is by far the most important environmental risk factor for the development of COPD. However, not all smokers develop COPD, suggesting that genetic factors that increase the susceptibility to the disease are also important in the pathogenesis of COPD [37]. So far, previous linkage analysis studies have identified many candidate genes that are associated with the development of COPD, such as PI MZ α-1 antitrypsin gene [38], matrix metalloproteinase 12 (*MMP12*) [39], transforming growth factor β-1 (*TGFB*_*1*_) and glutathione S-transferase Mu 1 (*GSTM1*) [40]. Due to recent developments in high-throughput genotyping screening techniques, genome-wide association study (GWAS) could be performed in a relatively rapid and inexpensive manner [41, 42]. By the case–control basis, this study enables to examine a genome-wide panel of single-nucleotide polymorphisms (SNPs) in association with phenotypes of interest. Recently, using this approach, Ota et al. demonstrated that the polymorphism in the *KCNA3* gene, which encodes Kv1.3, was associated with the susceptibility to autoimmune pancreatitis, in which T cell-mediated over-activation of cellular immunity is responsible for the pathogenesis [43]. The similar approach could be applied to detect the genetic variants of the human Kv1.3 gene in association with the phenotypes of COPD, such as the severity of the disease, the results of lung function tests and the findings of chest CT scanning [42]. Such genetic variance could also be linked with the epidemiological factors of COPD, including its prevalence, morbidity, mortality and comorbidity rates [44, 45].

## Therapeutic implications of targeting Kv1.3-channels in the treatment of COPD

Kv1.3-channels can be pharmacologically targeted by a variety of blockers of the channels [11, 46]. Additionally, in our series of patch-clamp studies, commonly used drugs, such as calcium channel blockers (CCBs) [25, 27], macrolide antibiotics [24], HMG-CoA reductase inhibitors (statins) [26] and nonsteroidal anti-inflammatory drugs (NSAIDs) [23], also effectively inhibited the Kv1.3-channel currents in thymus-derived murine lymphocytes. Of note, benidipine, one of the dihydropyridine-type CCBs, which most strongly and persistently inhibited the channel currents [25], actually suppressed the proliferation of kidney lymphocytes, and thus ameliorated the progression of renal fibrosis [47]. In this regard, in addition to using the previously developed selective blockers for the channels [48–50], the use of CCBs, macrolide antibiotics or statins could also potentially be useful in the treatment of COPD (Fig. 1).

In recent systematic review, statins were actually shown to prevent the exacerbation of COPD and reduce the risk of mortality in these patients [51, 52]. This could be explained by their pleiotropic effects, including anti-inflammatory and immunomodulatory effects, which was demonstrated by the significant decrease in systemic inflammation markers such as C-reactive protein and IL-6 [53, 54]. Additionally, statins were demonstrated to regulate the balance between Th1 and Th2 cells by inhibiting the Th1 development but augmenting the Th2 development of CD4^+^ cells [55]. In addition to statins, NSAIDs, such as ibuprofen or indomethacin, were also effective in patients with COPD, since these drugs ameliorated the production of bronchorrhea in these patients by reducing the concentration of prostaglandin E_2_ in their sputum [56]. Using lymphocytes isolated from patients with COPD, we would apply the patch-clamp recording technique and examine the production of pro-inflammatory cytokines from lymphocytes. Thus, we could reveal how the statins or NSAIDs-induced inhibition of Kv1.3-channels affects the activation of lymphocytes in vitro. To further reveal the therapeutic efficacy of these drugs in the pathogenesis of COPD, we would administer them to the experimental animal models of COPD [30, 31]. Then we would quantify the production of pro-inflammatory cytokines in the airways or alveolar parenchyma, or histopathologically analyze the degrees of bronchial inflammation, small airway fibrosis and alveolar destruction.

Recently, the continuous use of low-dose macrolide antibiotics was actually shown to be an effective treatment for COPD [57–59]. In randomized, double-blinded and placebo-controlled studies, Suzuki et al. and Seemungal et al. demonstrated that long-term erythromycin therapy was beneficial in the prevention of COPD exacerbations, which eventually contributed to the reduced number of hospitalizations [60, 61]. In other prospective, parallel-group and placebo-controlled studies, Blasi et al. and Albert et al. additionally revealed that the continuous administration of azithromycin decreased the rate of COPD exacerbations, and thus improved the quality of life of these patients [62, 63]. Besides, in some case reports, macrolide antibiotics, such as clarithromycin, ameliorated the extra-pulmonary symptoms of *Mycoplasma pneumonia* infection, such as cold agglutinins-induced hemoglobinuria, which are primarily attributable to the increased activity of T-lymphocytes [64, 65]. Concerning the therapeutic efficacies of macrolide antibiotics apart from their anti-microbial properties, several in vitro studies revealed that macrolide antibiotics, such as clarithromycin and azithromycin, suppress the production of pro-inflammatory cytokines from T-lymphocytes and thus exert immunomodulatory effects [66, 67]. In our recent patch-clamp study using murine thymocytes, clarithromycin markedly suppressed the Kv1.3-channel currents in a dose-dependent manner, suggesting its higher potency an useful channel inhibitor [24]. In T-lymphocytes, the Kv1.3-channels trigger calcium influx, which is necessary for IL-2 synthesis [11, 68], and channel blockade by highly selective inhibitors, such as margatoxin and ShK-Dap^22^, actually represses the immune response in the cells [28, 69]. Therefore, the potency of macrolide antibiotics as a Kv1.3-channel inhibitor was likely to be responsible for their immunomodulatory properties that have recently been demonstrated in the treatment of COPD [57–59].

## Conclusion

From our recent findings and the literature review, Kv1.3-channels are very likely to be overexpressed or over-activated in T-lymphocytes isolated from patients with COPD, which may contribute to the development or progression of the disease. Based on this, we suggest novel therapeutic implications of potentially useful Kv1.3-channel inhibitors, such as CCBs, macrolide antibiotics, statins and NSAIDs, in the treatment of COPD.
